# Supplementary material for: Biomphalaria pfeifferi infected with Schistosoma spp. in Kakamega and Bungoma counties, western Kenya confirms autochthonous transmission of intestinal schistosomiasis
Source: PLoS Negl Trop Dis. 2026 May 4;20(5):e0014276. doi: 10.1371/journal.pntd.0014276 (PMC13160442; doi:10.1371/journal.pntd.0014276)
Supplement: Attachment — Submitted filename: Response_reviewers__04032026.pdf [file pntd.0014276.s003.pdf]

KENYA MEDICAL RESEARCH INSTITUTE

Centre for Global Health Research, P. O. Box 1578-40100, KISUMU-KENYA
Tel: (254) (057) 2022923/24, Email: cghr@kemri.org Website: www.kemri.org

March 02, 2026

Editor-in-Chief,

PLOS Neglected Tropical Diseases:

Responses to Reviewer Comments for Manuscript: PNTD-D-25-01648R1 - Presence and infectivity of intermediate hosts of Schistosomiasis in Kakamega and Bungoma counties, western Kenya: Confirmation of autochthonous transmission"

We are pleased to submit our responses to reviewer comments for the aforementioned manuscript. The comments were very helpful and valuable in strengthening the manuscript.

We have provided our itemised responses to each point raised by the editor and reviewer(s). The responses are highlighted in yellow.

All authors have approved the submission of the revised manuscript.

We hope the responses meet your satisfaction and look forward top your feedback.

Sincerely,

Maurice Odiere, PhD
Corresponding author
Neglected Tropical Diseases Unit | Centre for Global Health Research,
Kenya Medical Research Institute (KEMRI)
Member, African Research Network for Neglected Tropical Diseases (ARNTD) Board | **https://arntd.org/**
W: **https://www.kemri.go.ke/**
Tel: +254 20 2726781/+254 20 2722541
Email: modiere@kemri.go.ke / mauriceodiere@gmail.com

Journal Requirements:

1) Please ensure that the CRediT author contributions listed for every co-author are completed accurately and in full.
At this stage, the following Authors/Authors require contributions: Maurice R Odiere. Please ensure that the full contributions of each author are acknowledged in the "Add/Edit/Remove Authors" section of our submission form.
The list of CRediT author contributions may be found here: https://journals.plos.org/plosntds/s/authorship#loc-author-contributions

The author contributions for Maurice R Odiere have been added.

2) Please provide an Author Summary. This should appear in your manuscript between the Abstract (if applicable) and the Introduction, and should be 150-200 words long. The aim should be to make your findings accessible to a wide audience that includes both scientists and non-scientists. Sample summaries can be found on our website under Submission Guidelines: https://journals.plos.org/plosntds/s/submission-guidelines#loc-parts-of-a-submission

AUTHOR SUMMARY

Recent mapping in Kakamega and Bungoma Counties of western Kenya showed many cases of intestinal schistosomiasis (bilharzia). However, it was unclear whether the transmission of the disease was taking place on site. To investigate this, a snail survey was conducted, because certain freshwater snails carry and release the parasite that infects people. The survey sampled water bodies near 42 primary school regions in 19 Wards where infection levels were high. Snails were collected, identified by species, and tested in the laboratory to see if they were releasing infectious parasite larvae. Out of 4,245 snails collected, most (about 79%) were Biomphalaria pfeifferi, the main carrier of Schistosoma mansoni. These snails were found at 36 out of 42 primary school regions sampled for snails, especially in streams and swamps. Importantly, 2.6% of them were actively shedding parasite larvae, meaning they were capable of infecting people. These findings confirm that schistosomiasis transmission is ongoing in these counties, not just a result of past exposure or imported cases. This study suggests that, in addition to treating people with medication, targeted snail control in high-risk areas could help stop transmission faster.

3) Please upload all main figures as separate Figure files in .tif or .eps format. For more information about how to convert and format your figure files please see our guidelines: https://journals.plos.org/plosntds/s/figures

All main figures have been attached as separate figures

4) Some material included in your submission may be copyrighted. According to PLOSu2019s copyright policy, authors who use figures or other material (e.g., graphics, clipart, maps) from another author or copyright holder must demonstrate or obtain permission to publish this material under the Creative Commons Attribution 4.0 International (CC BY 4.0) License used by PLOS journals. Please closely review the details of PLOSu2019s copyright requirements here: PLOS Licenses and Copyright. If you need to request permissions from a copyright holder, you may use PLOS's Copyright Content Permission form.

Please respond directly to this email and provide any known details concerning your material's license terms and permissions required for reuse, even if you have not yet obtained copyright permissions or are unsure of your material's copyright compatibility. Once you have responded and addressed all other outstanding technical requirements, you may resubmit your manuscript within Editorial Manager.
Potential Copyright Issues:

- Please confirm (a) that you are the photographer of Figure 2., or (b) provide written permission from the photographer to publish the photo(s) under our CC BY 4.0 license.

I confirm that I am the photographer of Figure 2.

- Figures 1 and 3. Please (a) provide a direct link to the base layer of the map (i.e., the country or region border shape) and ensure this is also included in the figure legend; and (b) provide a link to the terms of use / license information for the base layer image or shapefile. We cannot publish proprietary or copyrighted maps (e.g. Google Maps, Mapquest) and the terms of use for your map base layer must be compatible with our CC BY 4.0 license.
Note: if you created the map in a software program like R or ArcGIS, please locate and indicate the source of the basemap shapefile onto which data has been plotted.
If your map was obtained from a copyrighted source please amend the figure so that the base map used is from an openly available source. Alternatively, please provide explicit written permission from the copyright holder granting you the right to publish the material under our CC BY 4.0 license.
If you are unsure whether you can use a map or not, please do reach out and we will be able to help you. The following websites are good examples of where you can source open access or public domain maps:
* U.S. Geological Survey (USGS) - All maps are in the public domain. (http://www.usgs.gov)
* PlaniGlobe - All maps are published under a Creative Commons license so please cite u201cPlaniGlobe, http://www.planiglobe.com, CC BY 2.0u201d in the image credit after the caption. (http://www.planiglobe.com/?lang=enl)
* Natural Earth - All maps are public domain. (http://www.naturalearthdata.com/about/terms-of-use/).

Source of the basemap https://www.gadm.org/download_country.html

5) In the online submission form, you indicated that "Data will be made available on reasonable request". All PLOS journals now require all data underlying the findings described in their manuscript to be freely available to other researchers, either
1. In a public repository
2. Within the manuscript itself
3. Uploaded as supplementary information.
This policy applies to all data except where public deposition would breach compliance with the protocol approved by your research ethics board. If your data cannot be made publicly available for ethical or legal reasons (e.g., public availability would compromise patient privacy), please explain your reasons by return email and your exemption request will be escalated to the editor for approval. Your exemption request will be handled independently and will not hold up the peer review process, but will need to be resolved should your manuscript be accepted for publication. One of the Editorial team will then be in touch if there are any issues.

All the data are contained withing this manuscript

6) Please amend your detailed Financial Disclosure statement. This is published with the article. It must therefore be completed in full sentences and contain the exact wording you wish to be published.
- State the initials, alongside each funding source, of each author to receive each grant. For example: "This work was supported by the National Institutes of Health (####### to AM; ###### to CJ) and the National Science Foundation (###### to AM)."
- State what role the funders took in the study. If the funders had no role in your study, please state: "The funders had no role in study design, data collection and analysis, decision to publish, or preparation of the manuscript.".
If you did not receive any funding for this study, please simply state: u201cThe authors received no specific funding for this work.u201d

Funding: This work was supported by The END Fund through the Deworming Innovation Fund (DIF), Award Number: KEN-004-AMRK. The funders had no role in study design, data collection and analysis, decision to publish, or preparation of the manuscript.

Reviewers' Comments:

Reviewer's Responses to Questions

Key Review Criteria Required for Acceptance?

As you describe the new analyses required for acceptance, please consider the following:

Methods

-Are the objectives of the study clearly articulated with a clear testable hypothesis stated?

-Is the study design appropriate to address the stated objectives?

-Is the population clearly described and appropriate for the hypothesis being tested?

-Is the sample size sufficient to ensure adequate power to address the hypothesis being tested?

-Were correct statistical analysis used to support conclusions?

-Are there concerns about ethical or regulatory requirements being met?

Reviewer #1: See Summary and General Comments

Reviewer #2: 1. The objectives of the study were clearly stated but not hypothesis was not defined.

We hypothesized that (i) snail vectors of *Schistosoma* spp. are present in in the study areas and (ii) that the prevalence of *Schistosoma* infection among host snails is greater than zero, providing evidence of autochthonous transmission of schistosomiasis in the study areas.

2. The methodology was detailed enough but no specific study design was stated. This was a cross-sectional survey and the info has been added.

3. The target population was well described and detailed for the methodology.

4. The sample size was purposive and large enough.

5. The statistical analysis used correctly support the conclusions.

6. Ethical approval was properly obtained.

Reviewer #3: The objectives of the study were clearly articulated and the methodology is okay.

Results

-Does the analysis presented match the analysis plan?

-Are the results clearly and completely presented?

-Are the figures (Tables, Images) of sufficient quality for clarity?

Reviewer #1: See Summary and General Comments

Reviewer #2: 1. The analysis presented tallies with the data analysis.
2. The results are clearly and completely presented.

3, The tables and figures are clear enough except for table 5.

It is not clear to us what the specific concern with Table 5 is. From our end, we have tried to present the results in Table in the best way possible. We have made some minor edits to the last column label to enhance clarity.

Reviewer #3: The analysis presented matched the analysis plan, however the results need to be clearly presented in some sections.

We have made edits throughout the Results section to enhance clarity.

Conclusions

-Are the conclusions supported by the data presented?

-Are the limitations of analysis clearly described?

-Do the authors discuss how these data can be helpful to advance our understanding of the topic under study?

-Is public health relevance addressed?

Reviewer #1: See Summary and General Comments

Reviewer #2: 1. The conclusion supports the data presented.
2. The limitations of the study were clearly stated.

3. The manuscript clearly explained and effectively related the usefulness of the data collected to better understand the topic. It also proffered recommendations for the advancement of the field of study.

4. The public health importance of the study was well stated and properly related.

Reviewer #3: The conclusions are relevant and the limitations of the study was clearly stated.

Editorial and Data Presentation Modifications?

Use this section for editorial suggestions as well as relatively minor modifications of existing data that would enhance clarity. If the only modifications needed are minor and/or editorial, you may wish to recommend “Minor Revision” or “Accept”.

Reviewer #1: See Summary and General Comments

Reviewer #2: Title: Kindly ensure that schistosomiasis is written in lowercase. The Title has been revised and no longer contains the word schistosomiasis. The revised titled reads "*Biomphalaria pfeifferi* infected with *Schistosoma* spp. in Kakamega and Bungoma counties, western Kenya confirms autochthonous transmission of *Schistosoma mansoni*."

Schistosoma mansoni, Schistosoma haematobium and other scientific names included in the manuscript should be italicized. All scientific names have been italicized.
Introduction:
The third paragraph, include reference (s) in "Three main endemic...............respectively". A reference has been included.

Materials and Methods
Kindly change the "C" in Kakamega County and Bungoma County to lowercase. The "C" in County has been changed to lowercase across the manuscript.

Snail sampling: Include the number of personnel per team. The teams comprised of 5 personnel. This information has now been included.

Result
Kindly rephrase for the purpose of clarity "A total of 4,245 snails were collected across the 42 sites, with all but one yielding snails". This has been rephrased to read "A total of 4,245 snails were collected across the 42 primary school regions, with all but one school region yielding snails" for clarity.

Table: How the proportion of snails (%) was gotten is not clear, kindly include an explanation in statistical analysis.

We have added text below the Table 1 to clarify how the proportion of snails (%) was derived. Specifically, we have included the text below:

[$] *Relative species abundance per county is derived from number of snails by species divided by the total number of snails collected in each county (n = 2711 for Kakamega; n = 1534 for Bungoma).*

[β] *Overall Relative abundance is derived as the number of snail species for the two counties divided by the total number of snails collected (n = 4245).*

Discussion
This point is not applicable to this study because a single season was observed – "Our findings corroborate the pattern observed for Bulinus spp. in Iringa, Tanzania, where Schistosoma spp. cercarial emergence was higher during the dry season [57],............ (presumably when temperatures are lower)".

I suggest deleting this statement because it does not reflect the objective of the study, which is not season based – "Elsewhere, Gouvras and others [31] observed that shedding by Biomphalaria did not show any variation between dry and rainy seasons. We appreciate this comment. However, the point we are making with the argument is not comparison across season, but the context of higher shedding of cercariae during the drier periods, and showcasing other studies that gave demonstrated the same.

Reviewer #3: Minor Revision

Summary and General Comments

Use this section to provide overall comments, discuss strengths/weaknesses of the study, novelty, significance, general execution and scholarship. You may also include additional comments for the author, including concerns about dual publication, research ethics, or publication ethics. If requesting major revision, please articulate the new experiments that are needed.

Reviewer #1: Reviewer summary

This manuscript presents a relevant and timely study addressing the distribution and infection status of intermediate snail hosts of Schistosoma in two counties of western Kenya, contributing valuable local-scale data to our understanding of transmission dynamics outside the Lake Victoria basin that is well described in other manuscripts. The paper is well written, with little to no grammatical errors, and generally well structured, and the fieldwork represents an applaudable and important effort in mapping potential foci of autochthonous transmission in areas where only human prevalence data is available. However, in its current form, the manuscript requires substantial revision before it can be considered for publication. Several essential aspects of data presentation, analytical clarity, and methodological transparency need improvement to ensure the results are interpretable, reproducible, and robust. My comments below are intended to be constructive and supportive of the authors' work and provide guidance on how to strengthen the manuscript to meet the standards of PLOS Neglected Tropical Diseases.

General comments on the manuscript content

Only a suggestion – but the term snail 'vectors' is increasingly being used, and could be used throughout this manuscript (such as suggested in the title). This is well noted and we have adapted as suggested in the text.

Cercariae shed from snails are being presumed as S. mansoni cercariae, can the authors convincingly rule out the possibility that cercariae could not be S. rodhaini in this locality? My knowledge of S. rodhaini distribution outside of the Lake Victoria shores is somewhat patchy (as

is the literature), but after doing a bit of further research, what I can say is that in two publications from Saoud (1966) - https://www.cambridge.org/core/journals/journal-of-helminthology/article/abs/on-the-morphology-of-schistosoma-rodhaini-from-kenya/462782B093093D8CA10BE7921BEF51F2 - AND - https://www.cambridge.org/core/journals/journal-of-helminthology/article/abs/susceptibility-of-some-planorbid-snails-to-infection-with-schistosoma-rodhaini-from-kenya/CE0289C35F756E7F7B3A998613B3A8E4 they reference work by Nelson (1962) in chapter 7 of the Ciba Foundation Symposium - Bilharziasis (a piece of work I cannot get hold of unfortunately) that they successfully infected Biomphalaria pfeifferi nairobiensis (now synonymised to B. pfeifferi) with S. rodhaini isolates from Kisumu region. Saoud then in the second publication described the use of this S. rodhaini isolate to infect snails in their lab, and also got a 37% infection rate of exposed B. pfeifferi. See also Brown (1994) Freshwater Snails of Africa – under B. pfeifferi description for further information and references on this species compatability with mammalian schistosomes. All in all, I think here it would be great to mention that some of these untyped schistosome infections may be *S. rodhaini* and not *S. mansoni*, but that is still to be established in future studies. The authors could even conduct schistosome targeted PCRs from snail tissue extracts of the samples collected, and therefore still type species given that it seems as though cercariae were not collected.

We appreciate this comment and the reflections therein. We acknowledge that whereas molecular confirmation of cercariae for *S. mansoni* would have strengthened our findings, we note also that standard identification keys based on cercaria morphology are a recommended and acceptable method for identification of cercariae. The reported *S. mansoni* infections in school-age children in these areas further corroborates the cercariae identified as those of *S. mansoni* and not *S. rodhaini*. Furthermore, to our understanding, *S. rodhaini* primarily infects rodents, and has a predominantly nocturnal or twilight emergence pattern, which makes it highly unlikely that the cercarie were of *S. rodhaini.*

“Infectivity” is used throughout the manuscript to refer to the number of snails found infected. I think this terminology should be changed to an alternative since the definition of infectivity, see: https://en.wikipedia.org/wiki/Infectivity
“In epidemiology, infectivity is the ability of a pathogen to establish an infection. More specifically, infectivity is the extent to which the pathogen can enter, survive, and multiply in a host. It is measured by the ratio of the number of people who become infected to the total number exposed to the pathogen.”
To apply this term to snails it would mean to say that one is testing the number of snails that get infected following exposure, but the snails collected in this study may not necessarily have been exposed before. It would be better to just use the commonly used term of ‘prevalence’ when discussing the number of snails found infected with parasites. The term “infectivity” had been used in a generic sense in our manuscript. We have considered the view and have revised the text from “infectivity” to “prevalence” and/or “infection of *S. mansoni*” where applicable across the manuscript as suggested.

I mention this multiple times in the line by line comments below, but not in all instances. The word site/school is used interchangeably throughout the manuscript to represent the area surveyed for snail sampling sites. This makes the manuscript quite complicated to follow since sometimes the snail sampling sites are also referred to as sites, or points, or human water contact sites… Please ensure consistency and help the reader to follow. I would suggest calling

the area sampled around the school that prevalence data is recorded from a previous study the ‘school region’, and then each snail sampling site a ‘human-water contact site’ or ‘snail sampling site’. This is well noted and adopted as suggested. For clarity, schools have been renamed “school region” and sampling sites as “snail sampling sites”.

A supplementary dataset needs to be provided with this study that includes all sampling data per snail sampling site. This should include all variables recorded (from GPS of human water contact points, snail species and abundance of each found, to the human activities reported at each). This will make the study more citable, reproducible and the data available for others to use in meta-analysis and such, or when considering transmission control in the area. Providing the full dataset broken down by each site would then allow for the ‘Availability of data and materials’ statement to ring true.

The dataset that includes sampling data for the sites has now been included as Supplementary material.

The methodology of how many sites were sampled per school region is not sufficient to make this study reproducible. The current explanation given is:
“In each site/village, water bodies including canals, rivers, streams, ponds, swamps/marshes and dams (Fig. 2) where there was evidence of human-water contact activities were selected for snail sampling with the help of local community health promoters (CHPs).”
However, there is no measure on how far away from the schools, sites are sampled, or how many sites per region? Was a radius around the school chosen for sampling within? Was it time limited per day which dictated how many sites could be sampled per school? Please clarify this to establish how extensively regions were sampled.

Line by line comments

Title
The title is somewhat misleading in my opinion.

1. Snails are intermediate hosts of the parasite Schistosoma spp., not the disease schistosomiasis.

This is noted. The title has been revised to read “*Biomphalaria pfeifferi* infected with *Schistosoma* spp. in Kakamega and Bungoma counties, western Kenya confirms autochthonous transmission of *Schistosoma mansoni*”.

2. The term infectivity, to me, seems to suggest that the study is practically testing the infectivity / compatibility of snail hosts to schistosomes, but actually, the study is measuring the prevalence of schistosome infected snails collected. We appreciate this feedback, and have now revised the Title to read “*Biomphalaria pfeifferi* infected with *Schistosoma* spp. in Kakamega and Bungoma counties, western Kenya confirms autochthonous transmission of *Schistosoma mansoni*”

3. Although the study does find Bulinus globosus, it does not find any infected snails or relate back to human data on Sh. I suggest the title therefore more directly refers to the Biomphalaria

pfeifferi species which is associated with the infected snail findings and past human prevalence data.

We have revised the Title as indicated in (2) above to be more direct and relevant to the findings.

4. As per my general comment above, as no molecular data truly confirms the cercs are S. mansoni, can one be confident to use the species name in the title?

I would suggest a title change to something more relevant to the study, like:
Biomphalaria pfeifferi infected with Schistosoma spp. in Kakamega and Bungoma counties, western Kenya, implies autochthonous transmission of Schistosoma mansoni

We very much appreciate this comment from the reviewer and have revised the title to read "*Biomphalaria pfeifferi* infected with *Schistosoma* spp. in Kakamega and Bungoma counties, western Kenya confirms autochthonous transmission of *Schistosoma mansoni*".

Regarding the recommendation to provide confirmation using molecular identification of a subset of the collected snails, we acknowledge this and not that this was not possible due to limited resources among other factors. Since molecular identification is not possible, we have acknowledged this as a limitation of the study. Specifically, we have added the text "identification of *S. mansoni* cercariae was based on morphological characteristics and was not confirmed by molecular techniques. We note however that while molecular confirmation of cercariae for *S. mansoni* would have strengthened our findings, standard identification keys based on cercaria morphology [29, 30] are a recommended and acceptable method for identification of cercariae. The reported *S. mansoni* infections in SAC in the study areas further corroborates the cercariae identified as those of *S. mansoni*".

We also wish to point out that *S. rodhaini* primarily infects rodents, and has a predominantly nocturnal or twilight emergence pattern, which makes it highly unlikely that the cercarie were of *S. rodhaini*.

Abstract
Clarify what the evidence is, can one simply not just say 'found individuals infected with Schistosoma mansoni in this area'. It is our considered view that our sue of strong evidence is technically sound as presented without necessarily going into specifics, and prefer to retain it as is. We have added "among school-age children" for clarity.

Change to 'follow-up malacological survey'. We prefer to retain it as "a follow-up malacological survey"

Change infectivity to 'prevalence of S. mansoni infections in snails was determined.' This has been revised as suggested.

Specify in abstract that each waterbody was surveyed one time during the study. We consider this to be too much detail in the abstract. We also wish to indicate that the number of times visited per waterbody is already included in the Methods section under "Study sites sampled" as stated "Each of the 284 sites sampled for snails in the primary school regions was visited once during the dry season (February 2025)".

It seems unusual to me to display confidence intervals on the total abundance of snails identified to each species (and number infected), since this is a direct measure of quantity rather than demonstrating variability around an estimate (like a mean). For the mean abundance – a range is given here as a presumably a standard deviation? Please correct me if I am wrong here. We wish to clarify that the 95% CI is indicated for the total number of snails is related to the relative proportion that is included in the parenthesis and not the absolute counts. The mean abundance is presented with the standard deviation (SD) for the mean.

It would be useful to know from the abstract at how many sites schistosome infected snails were found, and how widely spread across the study population. How about where infected snails were most likely to be found in terms of waterbody type? This all seems like information very relevant to the study that is not provided in the abstract. We appreciate this comment. We note however that we have indicated in the abstract that *B. pfeifferi* was found in 36 out of 42 primary school regions and also indicated that the numbers were highest in streams and swamps. We believe that is sufficient information to be covered in the abstract without going into too much detail.

Introduction
In the last sentence of the first paragraph:

“The existence of persistent hotspots of schistosomiasis transmission [8, 9] underscore the challenge of spatial heterogeneity in MDA success which is likely multifactorial, with snail distribution and abundance predicted to play a large role [11, 12].”

One could also mention the role of snail genetics associated with immunity and compatibility of the snails with S. mansoni, which could go some way to explaining presence of persistent hotspots. There are recent papers discussing exactly this on B. sudanica in Lake Victoria. This is true and correct. We have added “genetics” to the sentence and included a Reference. The revised sentence now reads “The existence of persistent hotspots of schistosomiasis transmission [8, 9] underscore the challenge of spatial heterogeneity in MDA success which is likely multifactorial, with snail distribution, abundance and genetics predicted to play a large role [11, 12, 13].”

“the type of snail species “ – there is not a ‘type’ of snail species per se, I would change to just “Delimiting the species of snail present..” This is noted and adopted as suggested.

“and the Lake Victoria basin area (mainly S. mansoni)..” – not clearly what ‘mainly’ refers to. Maybe state “S. mansoni with focal pockets of S. haematobium transmission” with a reference for clarity? We have revised this to instead read “where the main spp. is *S. mansoni*”.

“with a dearth of surveys in areas further west” – maybe make clear that by further west you mean western Kenya and not further west of Lake Victoria (which would be Uganda of course!). Maybe just state that dearth of surveys in counties of Western Kenya that do not border the lake shore? This is noted and corrected as suggested to read “with a dearth of surveys in counties of western Kenya that do not border the lake, including Kakamega and Bungoma”.

“For a long time, it was widely believed that there was no or very little schistosomiasis in the

four counties of western Kenya (Kakamega, Bungoma, Vihiga and Trans Nzoia)" – State when 'a long time' was exactly - what period were surveys conducted in the past before the data in 2021 was added? This comment is noted. The point we are making here is that historically it has been known that there was no or very little schistosomiasis in the four counties of western Kenya, and these areas were largely considered non-endemic. We prefer to state this as "The four counties of western Kenya (Kakamega, Bungoma, Vihiga and Trans Nzoia) have historically been considered non-endemic for schistosomiasis or with limited transmission".

"the presence, species type" – again there is no 'type' of species, it is just a species or not. Please check this terminology throughout and correct. We have deleted "type" as suggested.

Materials and Methods
Study area and population
The Figure 1 map could be improved if possible, showing neighbouring countries and regions other than Kenya for one, giving the sites relative position to Lake Victoria in the larger scale map would be nice to provide context for readers unfamiliar with the area. Also can the Yala and Isiukhu rivers be shown on there? There are many freely available freshwater body 'layers' / vectors available online that could be used (like shown in Figure 3!). Other features described in this paragraph would be really nice to be shown on the map too. other rivers, dams etc... Could the map also show ward boundaries since these are later discussed?
The picture is also quite pixelated in my reviewer copy, but this may be due to exporting and formatting. If difficult in R, I would suggest using freely available GIS software such as QGIS to make maps. Please improve in the revised copy.

A revised copy has been provided

For estimated population size, say what year this is for. We have qualified this by indicating that the estimated population is based on the 2019 census and provided a reference (KNBS, 2019).

Could the map also show relevant to the previous granular mapping work where human Schistosoma spp. infections are found? This would be helpful for putting the snail collections and gastropod infections in context. We have explored this suggestion, and it is our considered view that adding the locations where human infections occurred to the map would make the map too busy. Instead, we have opted to include the prevalence data (2021) for each of the primary school regions as an additional column in Table 3, which gives a much easier contextual comparison.

Why have the authors chosen to interchangeably use schools / sites to mean the same thing? This is somewhat confusing since snail localities are often referred to as 'sites'. This has been addressed as per the other Reviewer's comment above. We have opted to refer to the sites as "primary school regions" and the exact locations where snails were collected as "snail sampling sites" for clarity.

Study sites sampled
Text on site selection a bit confusing, suggestions:
"10 Wards with 22 sites were included in Kakamega and 9 Wards with 20
sites for Bungoma" – combine with the first sentence of paragraph when mentioned 19 sites

selected. The sentence has been moved to follow immediately after the first sentence of the paragraph where 19 Wards are mentioned for easy flow.

"The selected sites were among those included in the granular mapping..." – You already mentioned this in first sentence of the paragraph – combine these sentences for clarity. We acknowledge that this is a repetition since it is already stated in the opening sentence of the paragraph. We have deleted the sentence.

"Each of the 42 sites was visited once during the dry season (February 2025)." – This is where it gets confusing with the schools / sites definition. You visited multiple human water contact sites (rivers / streams) per 'site / school'. This sentence sounds like just 42 freshwater sites were visited. How many freshwater sites were actually surveyed across the 42 regions surrounding the schools selected I presume will come up in the results. We acknowledge that this was confusing to the readership. This has been addressed by including the snail sampling locations/sites visited as and also as in aforementioned response above. The revised sentence now reads "Each of the 284 sites sampled for snails was visited once during the dry season (February 2025)."

Also – how far outside the school region were sites sampled? Was a radius site around the school to determine how many sites sampled? Was every site within the ward sampled?? Was it limited by time to survey within a day to do as many as possible per school region? Currently this is not clear and the sampling strategy of how snail sites were identified and selected needs to be explained better. We did not set a radius around the school to determine how many sites to be sampled but rather worked with CHPs to identify the nearest main water bodies. We had stated in the manuscript under "Snail sampling" that "Working closely with the local CHP in charge of a village, an effort was made to identify the main water bodies at each selected primary school region, where several ideal sampling points (range: 1-7) were explored at each water body". The range of sampled points is now revised to 1-15. We had also stated that "A timed direct search method was employed, where at each sampling point, a total of 20 random scoops were made on every sampling occasion. The area/size per sampling point was approximately 5 m^2 whereas lengths of 10 metres along streams, rivers and canals were used. Sampling time was fixed at 30 minutes at each water body".

Can the sites in Figure 2 be linked directly to GPS coordinates or site names from the surveys? Is there a supplementary dataset that lists the exact GPS coordinates that were recorded at each snail sampling site and the snails collected from there? This data would be very useful for future reference. Yes, the sites in Figure 2 can be linked directly to GPS coordinates and site names from the surveys. We have now provided the additional datasets containing this information as supplementary datasets.

Snail screening for cercariae

Were any of the cercariae released from snails preserved in ethanol or another DNA capture media? No. We did not preserve any of the cercariae released from the snails in ethanol or another DNA capture media.

Statistical analysis

The 'sites' and 'snail sampling localities' is confusing in this sentence also:
"The mean B. pfeifferi snail abundance was computed as the total number of snails collected per site divided by the number of points sampled per site." – so snail collecting sites now called points? This has been rephrased to read "The mean *B. pfeifferi* snail abundance was computed as the total number of snails collected per primary school region divided by the number of snail sampling locations in that region".

and also here...: "number of snails collected at a site with the initial prevalence in SAC at the corresponding site". This has been revised to read "number of snails collected at a school region site with the initial prevalence in SAC at the corresponding region".

Results
Snail abundance

"A total of 4,245 snails were collected across the 42 sites" – but how many human water contact sites for snail collection surveyed?? This is more relevant to report in relation to the abundance. This has been revised to read "A total of 4,245 snails were collected across the 284 sampled sites in the 42 school regions".

"rich snail species diversity" – rich compared to what? Remove descriptives in results and save for discussion when comparing and contrasting to other studies. The phrase "rich snail species diversity" is meant to provide context to the broad range of snail species collected and not for comparison. We prefer to retain it as is.

"7 freshwater snails..." – change to "7 freshwater snail species". This has been changed to "7 freshwater snail species" as suggested.

"Not surprisingly, we did not find any Biomphalaria sudanica, a species mainly found and involved in transmission around Lake Victoria [19, 31]." – this sentence is for the discussion and not results. This has been revised to read "No *Biomphalaria sudanica* snails were found at any of the sampled locations".

Throughout the results discussing the sites compare to human prevalence – it is not clear how close these sites are to the schools surveyed or inhabitants infected with S. mansoni. As indicated in the methods, main water does ere selected around each of the primary school region. The objective was to provide context for the observed human infections based on water bodies that serve the catchment where majority if the children would be coming from. It was not in the design of the study to set a certain radius for water bodies, but rather ton get an idea of the likely sources of water where transmission might be occurring to explain the observed infections in humans.

How about providing results on the abundance of snails / species relevant to the type of water body sampled? This seems more biologically relevant rather than comparing snail abundances between county boundaries. EDIT: can see that this is brought in in a later section – it would make sense to include it all here in the snail abundance section. The comparison for snails samples by county was to get an idea of the geographic distribution. Snail abundance by type of water source is presented in the manuscript.

Table 1 – as mentioned earlier, it seems unusual to report 95% CI with overall abundance values but correct me if I am wrong. This has been addressed above. The 95% CI refers to the proportions indicated in parenthesis, and not the absolute abundance number.

Table 2 – could table 2 also include the number of snails that shed mammalian schistosome cercariae rather than any type of cercariae per ward. This is more relevant for the paper. The following table 3 is much bigger and therefore harder to determine exactly where the majorities of mammalian schisto infections were observed. The total number of snails that shed mammalian schistosome cercariae rather than any type of cercariae per ward. A column for data on number of snails that shed mammalian schistosome cercariae has been added in Table 2.

Spatial distribution of snails and Schistosoma cercarial shedding

Table 3 – it seems key to add in here how many snail sampling sites were actually surveyed per 'school'. This will have an impact on the snail abundance and number infected? Although given by ward in Table 2, it should be broken down further here by locality. The number of snail sampling sites/points per school region have been added in Table 3.

Is B. pfeifferi abundance noted in sites in the Nzoia or tributaries or? From Figure 3 map, it looks like many sites further away from Nzoia that have an abundance of Bp too? So what does this mean? Yes, *B.pfeifferi* is noted in sites around Nzoia, but also along its tributaries.

"The highest number of B. pfeifferi (605 snails) was recorded at Indangalasia site (Table 3)…" – but again is this a reflection on there being a higher number of snail collection sites that were surveyed in that region? The summaries of abundance per school region could be misleading if not put in context of the sampling effort at each one. The number of sampling sites have been included in Table 3 to provide context. The sampling effort was roughly the same at each site, though the number of sites varied by site. The number remains highest for Indangalasia even when we do a comparison by mean abundance (47 snails.)

Fig 3 – the figure caption for Figure 3 is somewhat misleading – since the 42 points is per school, and the abundance of Biomphalaria is summed from all the sites withing that region associated with the school – correct? So therefore "…abundance of Biomphalaria snails at each of the sampled points…" seems to suggest that there is a point on the figure per snail sampling site – but this is actually school locality. This is noted and has been corrected to read "Map showing the geographic distribution of the 42 primary school regions and abundance of *Biomphalaria* snails within the school regions in Kakamega and Bungoma counties".

"Prevalence was highest at the Kisembe primary.." – the wording of this sounds like talking about human prevalence but refers to snails. Make clear when referring to snail infection prevalence and human prevalence. Also perhaps state in text here that the 72% was from 25 snails as shown in Table 3. Note this is a really high number of snails found naturally infected from such a small number, and quite unusual. Please discuss such things in the discussion. Were snail rinsed before plating to avoid cross contamination from the pot snails grouped in from the collection site? This has been corrected in the text to read "Prevalence of *S. mansoni* infection in *B. pfeifferi* was highest at the Kisembe primary school site region (72%) in Bunyala

West Ward". We confirm that all snails were rinsed prior to plating and this ensured there was not cross contamination.

Table 4 – may as well add schistosome cercariae to this table. Schistosome cercaria has been added to the Table as suggested.

Distribution of snails of Biomphalaria pfeifferi snails among water bodies in Kakamega and Bungoma counties
The reporting of the results could be improved and made easier to follow. It is clear that most snails were collected from streams, reporting as the 'proportion by water body type' is somewhat confusing and non-sensical if you just sampled from more stream sites than any other and therefore this represent the majority of your collection (as clear in table 5). We have presented the data based on the main water bodies that were identified in the primary school regions. We have tried to address the potential bias that could arise as a result of oversampling of sites at a water body by presenting the relative abundance and also mean abundance taking into consideration the number of sites sampled.

"The mean B. pfeifferi abundance at the streams was higher compared to rivers (P = 0.005)." – is it not significantly higher than ponds also? Was highest mean abundance from swamps / marshes when counting all sites? The text really doesn't reflect much of what is presented in the table leaving the reader to interpret themselves. This is noted and has been revised to read "The mean *B. pfeifferi* abundance at the streams was higher compared to rivers (P = 0.005) and ponds (P = 0.005) (Table 5)"

Table 5 – The lowercase letters representing significant differences don't make sense too – so for 'a' – streams swamps and marshes are all significantly different to each other? Based on what statistical test? This should be detailed in the table caption even if elsewhere in the methods as all tables and figures should be interpretable as stand-alone items. The lowercase letter comparison is a standard way of showcasing statistical differences across groups. Streams and marshes share the same lower-case letter (a), so they are not statistically different as explained in the footnote. The statistical comparison for mean snail abundance among the different water bodies was done using the Kruskal-Wallis test with post-hoc Dunn's test as indicated in the Statistical analysis section.

Final column of table 5 should also be the 'Total number of snails… in each water body type'. This has been revised to read "Total No. of snails shedding *S. mansoni* cercariae in each water body type"

Human water contact activities
It is a missed opportunity here to not perform any analysis connecting human behaviour to snail presence / absence and infected Biomphalaria with schisto snail presence. Is this data stored and reported elsewhere? This should be in the supplementary dataset. This is noted. However, the context for collection human water contact activities was to confirm that indeed there was human interaction with the water bodies that were sampled to help in corroborating them as active sites where transmission might be occurring. The human water contact activities are included in the supplementary dataset that is now provided.

Discussion
The discussion is well written, however I expected it will be revised in a the next version of the manuscript following the comments above. This has been done.

Reviewer #2: The study is a very important one because it will add to the available knowledge that underscores the need to integrate snail control in tackling schistosomiasis, particularly in endemic developing countries. However, the credibility of the report would have been stronger if confirmation using molecular identification of a subset of the collected snails was carried out, rather than solely relying on putative morphological identification that is not reliable. We appreciate this comment. However, due to resources available, molecular confirmation was not possible. We note however that while molecular confirmation of cercariae for *S. mansoni* would have strengthened our findings, standard identification keys based on cercaria are a recommended and acceptable standard method for identification of cercariae. The reported *S. mansoni* infections in school-age children in the study areas further corroborates the cercariae identified as those of *S. mansoni*. We believe the study advances the knowledge of transmission dynamics for schistosomiasis in western Kenya by confirming that indeed transmission is taking place in those areas and the reported cases represent local transmission and not imported cases.

Reviewer #3: Presence and infectivity of intermediate hosts of Schistosomiasis in Kakamega and Bungoma counties, western Kenya: Confirmation of autochthonous transmission

This study was conducted to ascertain the presence of Biomphalaria snail intermediate hosts following evidence of intestinal schistosomiasis infections in the area. The study is important for understanding the transmission of schistosomiasis in the region. The manuscript is fairly well written and would benefit from improvements.
The authors should conduct grammar formatting to improve the flow of the manuscript. Repetitions should be avoided to make it concise. We have conducted grammar formatting and deleted texts/sentences that had been repeated to make the manuscript more concise.

Abstract
1. Nineteen Wards which had Schistosoma mansoni prevalence ≥10% were selected from Kakamega and Bungoma counties, from which 42 sites (schools) that had the highest prevalence of S. mansoni were used to identify proximal water bodies with human-water contact activities to sample for intermediate hosts
Modify statement-long complex statement. Meaning unclear. Break into two clear statements.

The statement has been broken into two for clarity. The revised statement now reads "Nineteen Wards with ≥10% prevalence of Schistosoma mansoni were selected from Kakamega and Bungoma counties. From these Wards, 42 primary schools with the highest prevalence of *S. mansoni* were used to identify nearby water bodies with human-water contact activities for sampling snail vectors".

Introduction
1. Whereas chemotherapy
Change to Though chemotherapy. This has been changed to "Although chemotherapy".

Methods

1. The study involved 42 sites (schools) purposively selected from 19 Wards with the highest prevalence of S. mansoni in school-age children (SAC) in Kakamega and Bungoma counties (Fig. 2)

Fig 2 is not correct. This is noted and has been corrected to Fig 1.

2. (range: 1-7)

It is not clear what this means. This refers to the number of sampling locations/sites on the various water bodies that were sampled in the study (n = 284). This has been revised to reflect the correct range of 1-15 sampling locations.

Results

1. Whereas the mean B. pfeifferi abundance was lower in sites with ≥10% S. mansoni prevalence

Remove "whereas". This has been deleted.

2. For instance, at Okanya site in Bungoma, one of the sites with <10% S. mansoni prevalence it was high (115 snails),

Restructure statement. What do you mean by site in Okanya site? Are yiu referring to a type of water body, a river, swamp etc? This has been revised in light of the comments from the other reviewers to provide clarity between schools and snail sampled sites. We have now revised the reference to schools to read "primary school region" and the places where snails were collected to read "snail sampling sites". As a result, the sentence has been revised to read "For instance, at Okanya primary school region in Bungoma, one of the regions with <10% S. mansoni prevalence it was high (115 snails), while the mean abundance was low (1 snail) in Mahola primary school region in Kakamega, one of the regions with ≥10% S. mansoni prevalence".

3. Whereas the mean B. pfeifferi abundance was lower in sites with ≥10% S. mansoni prevalence compared to sites with <10% S. mansoni prevalence ($Y = -1.54x + 3.91$, $P = 0.011$), it was also highly variable among sites. For instance, at Okanya site in Bungoma, one of the sites with <10% S. mansoni prevalence it was high (115 snails), while the mean abundance was low (1 snail) in Mahola site in Kakamega, one of the sites with ≥10% S. mansoni prevalence.

Where is the data supporting this statement?

We have added a reference to indicate the information is from Table 3. We have also added mean abundance (in parenthesis) in the column on "No. of B. pfeifferi" in Table 3.

4. Elaborate on Table 2. We have now provided some elaboration for Table 2. Specifically, we have added the statement "A total of 1,106 and 2,235 *B. pfefferi* snails were collected in Bungoma and Kakamega counties, respectively (Table 2). Maraka Ward in Bungoma County had the highest number of snails that shed mammalian schistosome cercariae (Table 2). In Kakamega county, the highest number of snails that shed mammalian schistosome cercariae was 18 and 16 for Bunyala West Ward and Lusheya-Lubinu Wards, respectively (Table 2)."

5. Spatial distribution of snails and Schistosoma cercarial shedding
The authors should remove the column “No. of B. globosus”, the No. of B. pfeifferi (+) for S. mansoni should be combined with that of “Prev of S. mansoni in B. pfeifferi” as n (%). of S. mansoni (+) B. pfeifferi, the CI limit should be placed in a single column and written as xx-yy. The discussion should centre on Biomphalaria. The data for B. globosus can be placed in a new table with data on cercaria shedding as well. This is noted. We have combined the column “No. of *B. pfeifferi* (+) for *S. mansoni*” and that of “Prev of *S. mansoni* in *B. pfeifferi*” as suggested. We have then deleted the column titled “Prev of *S. mansoni* in *B. pfeifferi*”. We have also merged the 2 columns (LCL and UCL) for 95% CI for Prevalence into one. However, we prefer to retain the column with so that Table 3 gives a complete one-stop summary of the 2 main snail species, and to also reduce the number of Tables in the manuscript.

6. Human-water contact activities Several human-water contact activities were observed and noted among the different water bodies sampled. The activities included swimming, bathing, and fetching water for drinking and laundry. Other observed activities were irrigation, fishing and sand harvesting.
Remove this section. We prefer to retain this section as it provides the context that confirms that there are human-water contact activities taking place, further supporting the inference and conclusion around active transmission in those areas.

Discussion
7. The reasons for absence of snails in some regions should be discussed. This has now been added in last section of the opening paragraph of the Discussion.

General comments
Conduct grammar formatting. There are many ambiguous statements in the manuscript. We have conducted grammar formatting throughout the manuscript to make it succinct.

PLOS authors have the option to publish the peer review history of their article (what does this mean?). If published, this will include your full peer review and any attached files.

If you choose “no”, your identity will remain anonymous but your review may still be made public.

Do you want your identity to be public for this peer review? For information about this choice, including consent withdrawal, please see our Privacy Policy.

Reviewer #1: **Yes:** Tom Pennance

Reviewer #2: No

Reviewer #3: No

[NOTE: If reviewer comments were submitted as an attachment file, they will be attached to this email and accessible via the submission site. Please log into your account, locate the manuscript record, and check for the action link "View Attachments". If this link does not appear, there are no attachment files.]

Figure resubmission:

While revising your submission, we strongly recommend that you use PLOS's NAAS tool (https://ngplosjournals.pagemajik.ai/artanalysis) to test your figure files. NAAS can convert your figure files to the TIFF file type and meet basic requirements (such as print size, resolution), or provide you with a report on issues that do not meet our requirements and that NAAS cannot fix.

After uploading your figures to PLOS's NAAS tool - https://ngplosjournals.pagemajik.ai/artanalysis, NAAS will process the files provided and display the results in the "Uploaded Files" section of the page as the processing is complete. If the uploaded figures meet our requirements (or NAAS is able to fix the files to meet our requirements), the figure will be marked as "fixed" above. If NAAS is unable to fix the files, a red "failed" label will appear above. When NAAS has confirmed that the figure files meet our requirements, please download the file via the download option, and include these NAAS processed figure files when submitting your revised manuscript.

Reproducibility:

To enhance the reproducibility of your results, we recommend that authors of applicable studies deposit laboratory protocols in protocols.io, where a protocol can be assigned its own identifier (DOI) such that it can be cited independently in the future. Additionally, PLOS ONE offers an option to publish peer-reviewed clinical study protocols. Read more information on sharing protocols at https://plos.org/protocols?utm_medium=editorial-email&utm_source=authorletters&utm_campaign=protocols
