# Supplementary material for: Biomphalaria pfeifferi infected with Schistosoma spp. in Kakamega and Bungoma counties, western Kenya confirms autochthonous transmission of intestinal schistosomiasis
Source: PLoS Negl Trop Dis. 2026 May 4;20(5):e0014276. doi: 10.1371/journal.pntd.0014276 (PMC13160442; doi:10.1371/journal.pntd.0014276)
Supplement: Attachment — Submitted filename: Response to Reviewers.pdf [file pntd.0014276.s004.pdf]

KENYA MEDICAL RESEARCH INSTITUTE

Centre for Global Health Research, P. O. Box 1578-40100, KISUMU-KENYA
Tel: (254) (057) 2022923/24, Email: cghr@kemri.org   Website: www.kemri.org

April 02, 2026

Editor-in-Chief,

PLOS Neglected Tropical Diseases:

Responses to Reviewer Comments for Manuscript: PNTD-D-25-01648R1 - *Biomphalaria pfeifferi* infected with Schistosoma spp. in Kakamega and Bungoma counties, western Kenya confirms autochthonous transmission of *Schistosoma mansoni*"

We are pleased to submit our responses to reviewer comments for the aforementioned manuscript. The additional comments are helpful and valuable in strengthening the manuscript.

We have provided our itemised responses to each point raised by the reviewer. The responses are highlighted in yellow.

All authors have approved the submission of the revised manuscript.

We hope the responses meet your satisfaction and look forward top your feedback.

Sincerely,

Maurice Odiere, PhD
Corresponding author
Neglected Tropical Diseases Unit | Centre for Global Health Research,
Kenya Medical Research Institute (KEMRI)
Member, African Research Network for Neglected Tropical Diseases (ARNTD) Board | https://arntd.org/
W: https://www.kemri.go.ke/
Tel: +254 20 2726781/+254 20 2722541
Email: modiere@kemri.go.ke / mauriceodiere@gmail.com

Additional Editor Comments:

We appreciate the authors' attention to detail in this revised version of the manuscript. The remaining comment from the reviewer is important, and we request the authors to clarify in the Methods section that the cercariae species cannot be distinguished morphologically between S. mansoni and S. rodhaini, and to discuss this more clearly in the limitations of the discussion section.

Thanks for the feedback. We have clarified in the **Methods section** that the cercariae species cannot be distinguished morphologically between *S. mansoni* and *S. rodhaini*. Specifically, we have added the statement "Based on the morphological criteria for identification of cercariae used in this study, the cercariae species could not be distinguished morphologically between *S. mansoni* and *S. rodhaini* or for any other Schistosoma sp.", under section on "**Snail screening for cercariae**".

Please also ensure that the data sets are included with the next submission. They are not currently visible with the revised submission.

We have now included the datasets.

Journal Requirements:

Please provide separate figure files in .tif or .eps format. For more information about figure files please see our guidelines:

Reviewer #1: Thank you to Odiere and colleagues for submitting the revised manuscript with accompanying comments on the revisions made. I am very happy with the changes made across the manuscript and would like to thank the authors for taking their time to do this. I am recommending that the manuscript be accepted, however I am submitting as minor revision needed I would like to ask that the authors reconsider my point on cercariae ID as I have detailed again further AND I also did not receive a supplementary dataset with this submission that was stated by the authors (see below).

Cercariae identification

We would like to reiterate a point from my initial comments, which we do still believe could be addressed better, regarding the identification of schistosome cercariae to the species level. In the rebuttal, the authors states that cercariae morphology is sufficient to identify schistosome cercariae, and also state this again in the revised discussion, however the guides used by Frandsen as cited by the authors, only allow for an identification of cercariae to the genus *Schistosoma*, and not the species level as cercariae morphology overlaps. See this direct quote from Frandsen page 199:

"Numerous species of the genus Schistosoma exist in Africa (see Table 2) and assignment of a *Schistosoma sp*. cercaria to the species level is for the non-specialist not possible on the basis of morphological criteria because morphological differences between the cercariae of the

different species are extremely small."

Therefore, all schistosome cercariae morphological identified using this guide (which the authors used) should be listed as *Schistosoma sp*. In the discussion, one can then make the point that it is likely that these are *S. mansoni* rather than *S. rodhaini* based on the associated epidemiology and, to some extent, the shedding pattern. On that note, I do agree with the authors that the shedding patterns and timings of cercariae emergence are a great proxy to make a better guess of schistosome species ID, and I would suggest that the authors please edit the methods section "Snail screening for cercariae" with appropriate citations (as given in the discussion) that support the timing of shedding the snails in the methods, even though the time the authors shed snails is not necessarily optimal for *S. mansoni* (as the authors discuss in the limitations section of the discussion), it is notable also that the time they shed the snails overlaps with that of *S. rodhaini* in previous studies, for example see:
Norton, A., Rollinson, D., Richards, L. et al. Simultaneous infection of *Schistosoma mansoni* and S. rodhaini in Biomphalaria glabrata: impact on chronobiology and cercarial behaviour. Parasites Vectors 1, 43 (2008). https://doi.org/10.1186/1756-3305-1-43

" S. rodhaini's entire shedding period extended from 1430–1030 hours, however shedding was at a low level (<50 cercariae per snail) for all apart from the peak 2 hour period (1830–2030 hours)."

The authors state in their ms that the shed from 14-16:00 hours, which overlaps with this period. I would suggest that this is specifically stated by the authors, and that at least some mention to the S. rodhaini species is mentioned in this manuscript, as it is currently not mentioned at all and I think doing so only strengthens this manuscript.

Overall, this will be a small edit in the methods as suggested above, changing S. mansoni to 'Schistosoma sp. cercariae' in the results, and then in the discussion bring together to discuss the shedding Biomphalaria as being S. mansoni.

We appreciate this additional feedback. To address the comment, we have included several edits to the manuscript as outlined below:

1. We have edited the **Title** to exclude mention of species *Schistosoma mansoni*. The revised Title now reads "*Biomphalaria pfeifferi* infected with *Schistosoma* spp. in Kakamega and Bungoma counties, western Kenya confirms autochthonous transmission of intestinal schistosomiasis".
2. We have made edits throughout the Methods, Results and Discussion sections of the manuscript to replace *S. mansoni* cercariae with "*Schistosoma* sp. cercariae" as suggested.
3. In the **Methods**, under section on "**Snail screening for cercariae**" we have added the paragraph paragraph below to accommodate the concerns reading *S. rodhaini*.

   "The plate was exposed to indirect sunlight for 2 hours (1400–1600 hours) to induce cercarial shedding, a logistically driven timeframe that falls outside the optimal 0800–1300 hours emergence period for *S. mansoni* cercariae in *Biomphalaria* snails in this region [14]. This timeframe also overlaps with that for *S. rodhaini*, known to occur in this setting [14]. Based on the morphological criteria for identification of cercariae used in this study, the cercariae species could not be distinguished morphologically between *S. mansoni* and *S. rodhaini* or for any other *Schistosoma* sp".

4. We have edited and revised the paragraph below in order to bring together the discussion regarding the shedding Biomphalaria as being *S. mansoni* in the **Discussion**.

   "A low proportion of *B. pfeifferi* (2.6%) in our study shed *Schistosoma* sp. cercariae, likely to be those of *S. mansoni*, although we cannot rule out cercariae of *S. rodhaini*, known to occur in this setting [14]. *S. mansoni* cercariae emerge diurnally, peaking at dawn and midday [14] when their putative hosts enter the water and are available for infection. *S. mansoni* and *S. rodhaini* simultaneously infect *Biomphalaria* snails [14, 46], with *S. rodhaini*'s entire shedding period extending from 1430–1030 hours [46], which overlaps with the shedding period in our study. However, based on the associated epidemiology (confirmed *S. mansoni* infections among SAC [27]) and, to some extent, the shedding pattern it is most likely that these were *S. mansoni* rather than *S. rodhaini*."

   We have also added the **reference** by Norton *et al.* 2008 on Simultaneous infection of *S. mansoni* and *S. rodhaini* in *B. glabrata* snails and updated all the References in the manuscript accordingly.

Supplementary material

I would also like to make clear that the supplementary material/dataset was not made available to me while performing this second round of review, and do not see it cited within the manuscript anywhere, and I also note that in the final part of the manuscript it states:
"Availability of data and materials
All data supporting the findings of this study are available within the manuscript."
Which is now not the case if supplementary data is also included… I hope that this dataset can be included in the final manuscript as it will be highly valuable.

We apologize for this omission. We have now provided the additional datasets containing this information as supplementary datasets.

We have updated the final part of the manuscript **Availability of data and materials** to state

"All data supporting the findings of this study are uploaded as supplementary information"
